# Persistence and adherence to levodopa adjunct medications in elderly patients with Parkinson’s disease: a retrospective cohort study using a Japanese claims database

**DOI:** 10.3389/fneur.2025.1560431

**Published:** 2025-04-10

**Authors:** Masahiro Nagai, Michinori Koebis, Kotaro Sasaki, Chizuru Kobayashi, Kasumi Daidoji, Takayuki Ishida

**Affiliations:** ^1^Department of Neurology and Clinical Pharmacology, Ehime University, Ehime, Japan; ^2^Medical Headquarters, Eisai Co., Ltd., Tokyo, Japan; ^3^Deep Human Biology Learning, Eisai Co., Ltd., Tokyo, Japan

**Keywords:** Parkinson’s disease, persistence, adherence, health insurance claims database, levodopa adjunct medication, MAO-B inhibitors

## Abstract

**Objective:**

We investigated treatment persistence and adherence for levodopa adjunct medications and their relationship with demographic factors in Japanese patients with Parkinson’s disease (PD).

**Methods:**

This longitudinal retrospective study used a Japanese health insurance claims database for levodopa adjunct medications in patients newly prescribed anti-PD drugs other than levodopa between December 2020 and November 2021. Patients with a PD diagnosis were included in this study, and 17 anti-PD drug cohorts were formed. The primary outcomes were treatment persistence and adherence over 1 year. Multivariate analysis was conducted to evaluate demographic factors associated with treatment persistence/adherence.

**Results:**

In total, 7,605 patients were included in this analysis, with a mean age of 77.2 years, and 43.6% were male. The 1-year treatment persistence rate was 44.8%. Median persistent treatment duration over 1 year was 270.0 days. Persistence rates ranged from 28.6 to 59.5% across the drug cohorts, and were highest for zonisamide (59.5%) followed by safinamide (55.8%). The proportion of patients with proportion of days covered ≥80% (good treatment adherence) was 96.9% in the all-drugs cohort and ≥ 90% in each drug-specific cohort. In the multivariate analysis, the factor most strongly associated with non-persistence was the number of concomitant anti-PD drugs (risk ratio [RR] 0.85 per 1 unit increase), with the exception of inpatient prescriptions (RR 0.75).

**Conclusion:**

More than half of the new anti-PD drugs added to levodopa were discontinued within 1 year, and adherence to treatment, as assessed by filling records, was extremely high in patients with PD, including the elderly population.

## 1 Introduction

Parkinson’s disease (PD) is a progressive neurodegenerative condition mainly characterized by motor symptoms (including bradykinesia, tremor, rigidity, and postural instability) and non-motor symptoms (including cognitive abnormalities, autonomic dysfunction, sleep disorders, pain, and other sensory disturbances) (1, 2). More than 80% of patients with PD in Japan are over 65 years of age (3, 4). Levodopa is the gold standard of treatment for PD. However, long-term levodopa treatment is associated with motor complications, such as wearing-off and dyskinesia onset. As the disease progresses, adjunctive therapy becomes necessary in addition to levodopa. In Japan, various types of adjunctive drugs for wearing-off are available, including dopamine agonists, monoamine oxidase B inhibitors, catechol-O-methyltransferase inhibitors, adenosine A2A receptor antagonists, and zonisamide.

In general, the efficacy and safety of pharmacological treatment are associated with both medication discontinuation and adherence. For appropriate drug treatment of PD, it is essential for patients to be actively involved in their treatment and adhere to taking the medications prescribed by their doctor. However, as with other chronic diseases, adherence to treatment is low in patients with PD, with non-adherence rates reported to vary between 10 and 67% (5). As the prevalence of comorbidities increases with age, older adults are more prone to polypharmacy, which can negatively affect treatment adherence and persistence. Poor treatment adherence can lead to worsening of patients’ symptoms and the need for additional medication, which also increases healthcare costs (6). Therefore, non-adherence to treatment is an important problem in the management of PD that warrants attention.

Studies conducted overseas have identified several risk factors for poor treatment adherence, including age, length of illness, cognitive decline, depression, inadequate symptom control, complexity of treatment including polypharmacy, and drug side effects (5, 7). In Japan, there have been few longitudinal studies on drug prescriptions for PD (8, 9) owing to a lack of traceable medical information databases that include the elderly population, and previous studies have not examined persistence and adherence to levodopa adjunct medications. However, with the implementation of the elderly healthcare system in 2008, people aged ≥75 years are now covered by this system (10), and the elderly health insurance database has become available for research use.

This study aimed to longitudinally analyze treatment persistence rates and adherence to PD medication (excluding ergot dopamine agonists, and focusing on levodopa adjunct medications), along with the relationships between treatment persistence/adherence and comorbidities and polypharmacy, in Japanese patients with PD, including the elderly, during drug treatment.

## 2 Materials and methods

### 2.1 Study design

This was a longitudinal descriptive epidemiological study using medical claims information data from a claims database (IQVIA Claims D, IQVIA Solutions Japan G.K.), derived from two Japanese National Health Insurance systems: the latter-stage elderly health insurance system for people aged ≥75 years, and the National Health Insurance system for people aged <75 years and not covered by another public health system. The database contains medical information such as diagnosis codes, prescription drugs, and procedure codes. Medical information can be tracked even for an individual who visits multiple medical facilities, provided that individual maintains membership in the same health insurance program. The data extraction period was from December 2019 to November 2022. The cohort entry date was defined as the date of the first filling of the drug under analysis (index drug) during the data extraction period from December 2020 to November 2021. Each case was followed up for up to 365 days from the cohort entry date. The study design is shown in Supplementary Figure 1.

Seventeen drugs with an indication for PD, in either oral or transdermal patch formulations (excluding levodopa combination products and ergot dopamine agonists), were included in the analysis as an index drugs (Supplementary Table 1). Ergot dopamine agonists are available in Japan, but their prescription rates are low (8). Furthermore, in the 2018 Japanese Society of Neurology Parkinson’s Disease Clinical Practice Guidelines, ergot dopamine agonists are not recommended as a first-line agent due to their risk of side effects (11). This study included patients who newly started treatment for PD with anti-PD drugs other than oral levodopa while prescribed oral levodopa. Accordingly, patients treated with ergot dopamine agonists were excluded in this study. As droxidopa is indicated for the treatment of PD in patients with Hoehn-Yahr stage 3 who exhibit the symptoms of freezing gait in Japan (12), patients treated with droxidopa were included in this study. Zonisamide is also indicated for the treatment of PD as an adjunct to levodopa in Japan (13).

Cohorts were formed for each newly filled drug on the date of cohort entry, and these were combined to form the all-drugs cohort. If the all-drugs cohort included patients with the same identification number, only data for the drug with the earliest new filling date were used. In the all-drugs cohort, when multiple drugs (among those listed in Supplementary Table 1) were filled at the date of cohort entry, the index drug was defined as the combination of multiple drugs.

The study protocol was approved by the Clinical Research Ethics Review Committee of Ehime University Hospital. The study was conducted in accordance with the Declaration of Helsinki and in compliance with the Ethical Guidelines for Life Sciences and Medical Research Involving Human Subjects. Informed consent was waived because an anonymized commercial database was used. This study was registered at the UMIN Clinical Trials Registry under the identifier number UMIN000052869.

### 2.2 Patients

To include patients who newly started treatment for PD with anti-PD drugs other than oral levodopa while prescribed oral levodopa, patients who met all of the following criteria were included in the study: (1) patients with a definitive diagnosis of PD (International Statistical Classification of Diseases 10th Revision [ICD-10] (14) code: G20) in the same month as the cohort entry date, (2) patients for whom oral levodopa was filled both on the cohort entry date and in the 180 days immediately preceding the cohort entry date (look-back period), (3) patients with no index drug filling in the look-back period, and (4) patients aged ≥40 years at the date of cohort entry. Patients who met any of the following criteria were excluded from the study: (1) patients who did not have any data recorded during the look-back period or for 365 days after the date of cohort entry and/or (2) patients with a diagnosis of a disease that needs to be differentiated from PD, including multiple system atrophy, progressive supranuclear palsy, basal ganglia degeneration, vascular Parkinson’s syndrome, normal pressure hydrocephalus, drug-induced Parkinson’s syndrome, and schizophrenia. ICD-10 codes for the diseases used in this study are shown in Supplementary Table 2.

### 2.3 Outcomes

The primary outcomes were treatment persistence (persistence rate, period) and treatment adherence. A gap period was defined as the number of days from the last day of filling the index drug to the next filling day. Treatment was considered “persistent” if the gap period was less than 30 days; otherwise, it was deemed discontinued. The 1-year treatment persistence rate was evaluated for the all-drugs cohort and each drug cohort. Adherence was evaluated using the proportion of days covered (PDC) (15), that is, “the number of days in the covered period/number of days from the first fill date to the date before the start date of the last covered period” within the persistent treatment period.

The covered period refers to the period covered by index drug medication, that is, from the fill date until the earlier of either of the following events: (1) the last date covered by the prescription or (2) the next fill date. For the all-drugs cohort, the covered period was defined as the period covered by multiple index drugs filled on the cohort entry date. We calculated this as the period during which the covered period of each drug overlapped, with days between each covered period considered the gap period.

The analysis of adherence was restricted to patients with at least two prescriptions filled for the index drug to reduce potential bias and include patients established as continuous users. The adherence measure used in this study was adopted from the Quality Alliance and the National Association of Specialty Pharmacy (15). The proportion of patients with good treatment adherence, defined as a PDC ≥80%, was evaluated.

An exploratory multivariate analysis was conducted to evaluate factors associated with good treatment persistence and adherence in the all-drugs cohort. Explanatory variables included age, sex, number of drugs filled, daily levodopa dose at cohort entry date, hospitalization during the follow-up period, concomitant drugs, and presence of comorbidities (depression, dementia, and liver failure/renal failure [Supplementary Table 2]) during treatment. The number of concomitant drugs for other diseases was defined as the number of drugs, excluding those for PD (Anatomical Therapeutic Chemical code N04), filled in the cohort entry date or the look-back period. These drugs were classified according to the Anatomical Therapeutic Chemical second level (pharmacological or therapeutic subgroup). Those with less than 14 prescription days were excluded.

If the number of days drugs were prescribed for at discharge was recorded as 1 day, it was imputed with the number of days until the date of next fill, with a maximum of 30 days. This is because the prescription period for drugs filled at discharge in the database used may have been registered as 1 days’ worth of prescription, which does not reflect the actual intended duration of treatment. As this process could be confounding, hospitalization prescription was added as an explanatory variable in the multivariate analysis.

Subgroup analyses were also conducted using the explanatory variables in the multivariate analysis. We also assessed management fees for guidance on intractable outpatient diseases during the follow-up period as a proxy for identifying patients with severe PD.

### 2.4 Statistical methods

Descriptive statistics were used to summarize patient demographic and clinical characteristics. The frequency and proportion of patients who continued treatment for 1 year per cohort and who achieved good treatment adherence (PDC ≥80%) were calculated. The duration of treatment and PDC were summarized using quartiles, minimum, and maximum values. In addition, Kaplan–Meier curves were plotted using the treatment discontinuation as an event to visualize changes in treatment persistence over time. To explore factors associated with 1-year treatment persistence and good treatment adherence (PDC ≥80%), multivariate analyses were conducted in the all-drugs cohort using a modified Poisson regression model (16). This study was descriptive in nature; no statistical tests for comparisons between drugs were performed. The confidence interval (CI) was calculated using a 95% confidence level.

A sensitivity analysis was conducted to evaluate the robustness of the study results. For sensitivity analysis 1, the threshold for the gap period to determine persistent status was changed from 30 days to 15 days, and the same analysis as the primary analysis was performed. For sensitivity analysis 2, the exposure washout window for the treatment included in inclusion criterion 3 was changed to the period from the beginning of data extraction (December 1, 2019) to the day before the cohort entry date, and the same analysis as the primary analysis was performed. For sensitivity analysis 3, we referred to the constant follow-up period rather than the persistent treatment period to calculate the denominator in the PDC, and the PDC was computed as the number of days in the covered period/number of days from the first filling day to the day before the start of the last covered period within the follow-up period. For sensitivity analysis 4, an analysis similar to the treatment adherence analysis was performed using the medication position ratio (MPR) instead of the PDC. MPR was defined as the total number of prescription days from the first filling day to the day before the start of the last covered period within the follow-up period/number of days from the first filling day to the day before the start of the last covered period within the follow-up period (15), and could be >1.0. Sensitivity analyses 3 and 4 were conducted as *post hoc* analyses.

R version 4.3.1 (R Foundation for Statistical Computing, Vienna, Austria) was used for multivariate analysis and plotting Kaplan–Meier curves. All other statistical analysis was performed using Python version 3.10.13 (Python Software Foundation, Wilmington, Delaware, US).

## 3 Results

### 3.1 Patient characteristics

In total, 7,605 patients were included in this analysis. The background characteristics of the study population are summarized in Table 1 and Supplementary Table 3. In the all-drugs cohort, the mean ± standard deviation (SD) age was 77.2 ± 7.0 years, 3,318/7,605 (43.6%) patients were male, the median (interquartile range [IQR]) levodopa equivalent dose was 450.0 (300.0–650.0) mg, and the proportions of patients prescribed 1, 2, and ≥ 3 anti-PD drugs were 46.9% (3,564/7,605), 27.5% (2,095/7,605), and 25.6% (1,946/7,605), respectively. Most background characteristics of the drug-specific cohorts were similar. However, the mean number of concomitant anti-PD drugs (ranging from 1.6 to 2.8) and median levodopa equivalent dose differed from cohort to cohort (ranging from 300 to 685 mg).

**Table 1:** Patient demographic and clinical characteristics.

Characteristics	Overall *n* = 7,605
Age, years, mean ± SD	77.2 ± 7.0
Male, *n* (%)	3,318 (43.6)
Daily levodopa dose, mg, median (IQR)^a^	300.0 (300.0–450.0)
Daily levodopa equivalent dose, mg, median (IQR)^a^	450.0 (300.0–650.0)
Number of anti-PD drugs, mean ± SD	1.9 ± 1.1
1, *n* (%)	3,564 (46.9)
2, *n* (%)	2095 (27.5)
≥ 3, *n* (%)	1946 (25.6)
Anti-PD drugs filled, *n* (%)	
Ergot dopamine agonists	91 (1.2)
Non-ergot dopamine agonists	4,422 (58.1)
Monoamine oxidase B inhibitors	3,167 (41.6)
Catechol-O-methyltransferase inhibitors	1,605 (21.1)
Anticholinergics	445 (5.9)
Others	3,546 (46.6)
Number of concomitant drugs other than anti-PD drugs, mean ± SD	5.2 ± 3.1
Charlson Comorbidity Index, mean ± SD	2.0 ± 1.9
Comorbidities, *n* (%)	
Depression	1,087 (14.3)
Insomnia	2,951 (38.8)
Dementia	1,478 (19.4)
Type 2 diabetes	2,531 (33.3)
Hypertension	4,428 (58.2)
Liver failure	1,185 (15.6)
Renal failure	424 (5.6)
Constipation	5,650 (74.3)
Pain	2,695 (35.4)
Deep brain stimulation, *n* (%)	98 (1.3)
Levodopa injection, *n* (%)	6 (0.1)
Hospitalization in persistent treatment period, *n* (%)	1,172 (15.4)
Claims of management fee for intractable diseases in persistent treatment period, *n* (%)	4,697 (61.8)

a Daily levodopa dose and daily levodopa equivalent dose were calculated for patients with no records of levodopa injection and deep brain stimulation in the baseline period. Daily levodopa equivalent dose was calculated excluding target anti-PD drugs newly filled at cohort entry date in each cohort.

IQR, interquartile range; PD, Parkinson’s disease; SD, standard deviation.

The numbers of patients in each drug-specific cohort were: pramipexole (immediate-release tablet), 329; pramipexole (sustained-release tablet), 345; ropinirole (immediate-release tablet), 52; ropinirole (sustained-release tablet), 429; ropinirole (transdermal patch), 1,358; rotigotine, 867; selegiline, 425; rasagiline, 932; safinamide, 876; entacapone, 635; opicapone, 920; trihexyphenidyl, 200; biperiden, 7; droxidopa, 574; amantadine, 459; istradefylline, 452; and zonisamide, 850 (Supplementary Table 4).

### 3.2 Persistence

**Figure 1:**
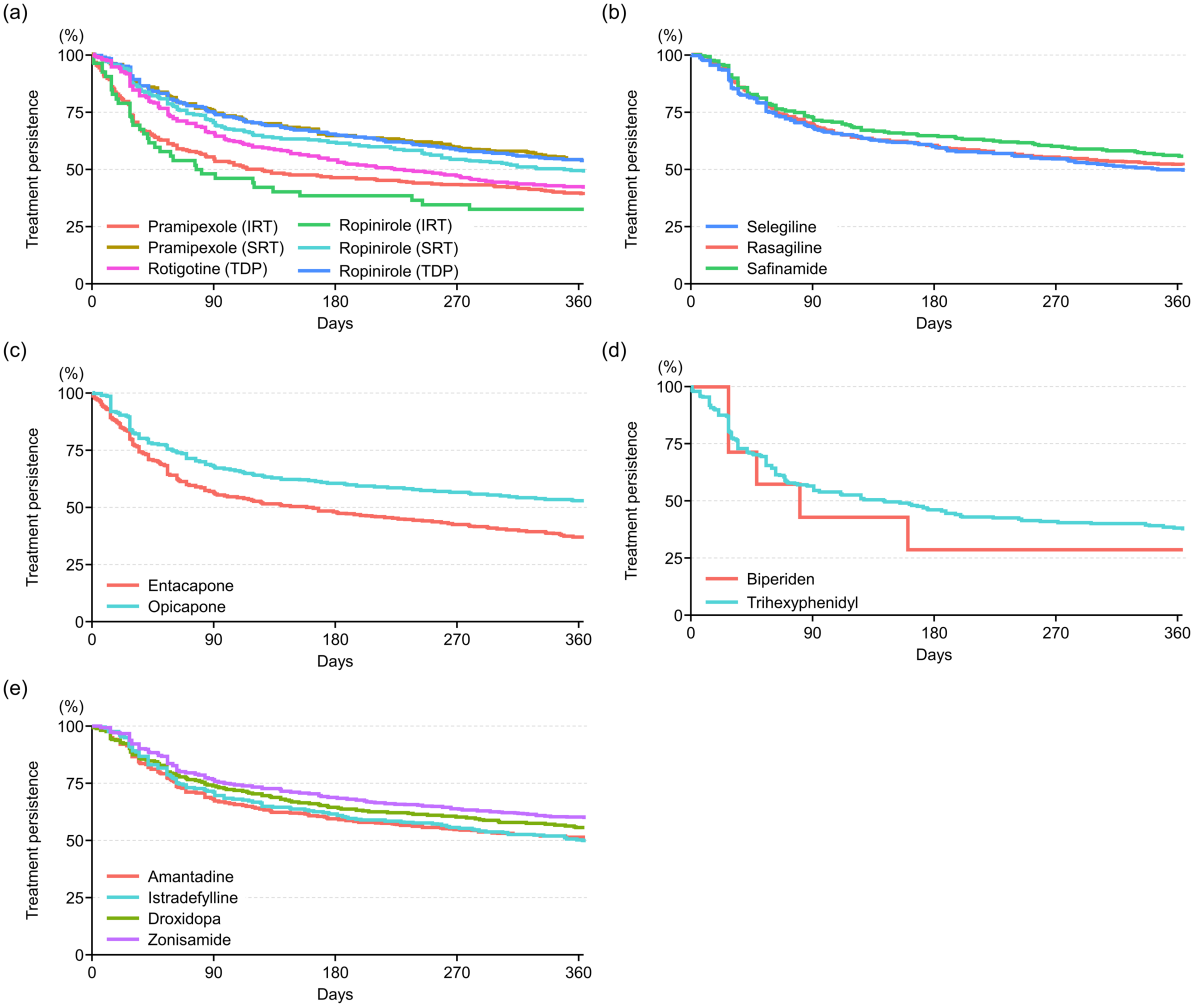
Kaplan–Meier plots of treatment persistence for anti-PD drugs: **(a)** non-ergot dopamine agonists, **(b)** monoamine oxidase B inhibitors, **(c)** catechol-O-methyltransferase inhibitors, **(d)** anticholinergics, and **(e)** others. IRT, immediate-release tablet; PD, Parkinson’s disease; SRT, sustained-release tablet; TDP, transdermal patch.

The persistence of anti-PD drugs in the all-drugs cohort and drug-specific cohorts are shown in Table 2, and Kaplan–Meier plots of treatment persistence for anti-PD drugs are shown in Figure 1. In most cohorts, treatment persistence rates tended to rapidly decrease within the first 90 days, and tended to gradually decrease thereafter. In the all-drugs cohort, the 1-year treatment persistence rate was 44.8% (3,410/7,605), with a median (IQR) persistent treatment period of 270.0 (56.0–365.0) days. Across the 17 drug-specific cohorts, the 1-year treatment persistence rates ranged from 28.6% (biperiden cohort) to 59.5% (zonisamide cohort), with median persistent treatment periods ranging from 79.0 to 365.0 days (IQRs 28.0–365.0 days). More than half of the patients on zonisamide (59.5%), safinamide (55.8%), droxidopa (55.6%), pramipexole (sustained-release tablet; 53.9%), ropinirole (transdermal patch; 53.9%), opicapone (52.7%), rasagiline (51.9%), and amantadine (51.0%) continued treatment for 1 year without experiencing a 30-day gap period (Table 2).

**Table 2:** Persistence of anti-PD drug treatment.

Treatment	*n*	Persistent patients, *n* (%)	Persistent treatment period (days)				
			Min	Q1	Median	Q3	Max
All drugs	7,605	3,410 (44.8)	1.0	56.0	270.0	365.0	365.0
Non-ergot dopamine agonists							
Pramipexole (immediate-release tablet)	329	128 (38.9)	1.0	28.0	126.0	365.0	365.0
Pramipexole (sustained-release tablet)	345	186 (53.9)	1.0	91.0	365.0	365.0	365.0
Ropinirole (immediate-release tablet)	52	17 (32.7)	1.0	28.0	79.0	365.0	365.0
Ropinirole (sustained-release tablet)	429	210 (49.0)	1.0	70.0	350.0	365.0	365.0
Ropinirole (transdermal patch)	1,358	732 (53.9)	1.0	90.0	365.0	365.0	365.0
Rotigotine (transdermal patch)	867	363 (41.9)	1.0	56.0	225.0	365.0	365.0
Monoamine oxidase B inhibitors							
Selegiline	425	210 (49.4)	1.0	60.0	346.0	365.0	365.0
Rasagiline	932	484 (51.9)	2.0	63.0	365.0	365.0	365.0
Safinamide	876	489 (55.8)	7.0	77.0	365.0	365.0	365.0
Catechol-O-methyltransferase inhibitors							
Entacapone	635	234 (36.9)	1.0	35.0	159.0	365.0	365.0
Opicapone	920	485 (52.7)	1.0	59.8	365.0	365.0	365.0
Anticholinergics							
Trihexyphenidyl	200	75 (37.5)	1.0	35.0	143.5	365.0	365.0
Biperiden	7	2 (28.6)	28.0	38.5	81.0	263.0	365.0
Others							
Droxidopa	574	319 (55.6)	1.0	84.0	365.0	365.0	365.0
Amantadine	459	234 (51.0)	1.0	62.5	365.0	365.0	365.0
Istradefylline	452	223 (49.3)	3.0	64.0	363.0	365.0	365.0
Zonisamide	850	506 (59.5)	2.0	98.3	365.0	365.0	365.0

Q, quartile; PD, Parkinson’s disease.

The subgroup analysis of persistence of anti-PD drugs is shown in Supplementary Table 5. The trends regarding differences in persistence rates by drug were similar across the subgroups. In particular, differences between drugs were more apparent in the levodopa ≥400 mg subgroup, the subgroup with concomitant anti-PD medications, and the subgroup with hospitalization during the follow-up period.

Table 3 shows the factors associated with persistence of anti-PD drugs in the all-drugs cohort. Age (risk ratio [RR] 0.99, 95% CI 0.99–1.00; *p* = 0.008), male sex (RR 1.07, 95% CI 1.02–1.12; p = 0.008), number of anti-PD drugs filled (RR 0.85 per 1 unit increase, 95% CI 0.81–0.88; *p* < 0.001), number of concomitant drugs filled (RR 1.01 per 1 unit increase, 95% CI 1.00–1.02; *p* = 0.030), prescriptions of anti-PD drugs during hospitalization (RR 0.75, 95% CI 0.69–0.81; *p* < 0.001), and depression (RR 0.90, 95% CI 0.84–0.98; *p* = 0.010) were found to be significantly associated with persistence of anti-PD drugs in the all-drugs cohort.

**Table 3:** Factors associated with persistence of anti-PD drugs in the all-drugs cohort.

	Persistent patients *n* = 3,369	Non-persistent patients *n* = 4,132	Risk ratio (95% CI)	*p*-value
Age, years, mean ± SD	77.2 ± 6.8	77.4 ± 7.0	0.99 (0.99–1.00)	0.008
Sex, *n* (%)				
Male	1,531 (45.4)	1740 (42.1)	1.07 (1.02–1.12)	0.008
Female	1838 (54.6)	2,392 (57.9)	reference	
Number of drugs filled, mean ± SD				
Anti-PD drugs	1.8 ± 1.0	2.0 ± 1.2	0.85 (0.81–0.88)	<0.001
Concomitant drugs	5.2 ± 3.1	5.2 ± 3.1	1.01 (1.00–1.02)	0.030
Daily levodopa dose at day 0, mg, median (IQR)				
Levodopa	300.0 (300.0–450.0)	350.0 (300.0–500.0)	0.98 (0.96–1.00)	0.120
LED of all anti-PD drugs	400.0 (300.0–600.0)	450.0 (300.0–699.0)	1.01 (1.00–1.03)	0.139
Prescriptions of anti-PD drugs during hospitalization, *n* (%)	393 (11.7)	747 (18.1)	0.75 (0.69–0.81)	<0.001
Comorbidities, *n* (%)				
Depression	441 (13.1)	628 (15.2)	0.90 (0.84–0.98)	0.010
Dementia	638 (18.9)	821 (19.9)	0.99 (0.93–1.06)	0.731
Liver failure/renal failure	640 (19.0)	827 (20.0)	0.94 (0.89–1.01)	0.082

CI, confidence interval; IQR, interquartile range; LED, levodopa equivalent dose; PD, Parkinson’s disease; SD, standard deviation.

### 3.3 Adherence

The results of PDC of anti-PD drugs to the last fill date in the persistent treatment period are shown in Table 4. The proportions of patients with PDC ≥80% (good treatment adherence) were 96.9% (5,152/5,318) in the all-drugs cohort and ≥ 90% (ranging from 90.0 to 100.0%) in each drug-specific cohort. In the subgroups analyzed, the proportion of patients with good treatment adherence ranged from 71.4 to 100.0% (Supplementary Table 6).

**Table 4:** PDC of anti-PD drug treatment to the last fill date in the persistent treatment period.

Treatment	*n*	PDC ≥ 80%, *n* (%)	PDC, %				
			Min	Q1	Median	Q3	Max
All drugs	5,318	5,152 (96.9)	7.1	96.0	99.7	100.0	100.0
Non-ergot dopamine agonists							
Pramipexole (immediate-release tablet)	227	207 (91.2)	12.5	94.0	99.1	100.0	100.0
Pramipexole (sustained-release tablet)	262	249 (95.0)	17.6	96.5	99.7	100.0	100.0
Ropinirole (immediate-release tablet)	30	27 (90.0)	7.1	95.2	98.9	100.0	100.0
Ropinirole (sustained-release tablet)	325	313 (96.3)	20.8	97.6	100.0	100.0	100.0
Ropinirole (transdermal patch)	1,015	1,007 (99.2)	54.0	97.4	100.0	100.0	100.0
Rotigotine (transdermal patch)	585	577 (98.6)	51.6	96.7	99.7	100.0	100.0
Monoamine oxidase B inhibitors							
Selegiline	304	299 (98.4)	45.7	96.4	99.7	100.0	100.0
Rasagiline	655	644 (98.3)	14.3	97.6	100.0	100.0	100.0
Safinamide	642	630 (98.1)	36.4	96.1	99.7	100.0	100.0
Catechol-O-methyltransferase inhibitors							
Entacapone	421	384 (91.2)	10.8	95.0	99.7	100.0	100.0
Opicapone	656	632 (96.3)	44.4	95.5	99.2	100.0	100.0
Anticholinergics							
Trihexyphenidyl	119	116 (97.5)	53.6	94.9	99.4	100.0	100.0
Biperiden	4	4 (100.0)	97.0	97.1	98.6	100.0	100.0
Others							
Droxidopa	445	436 (98.0)	17.5	95.8	99.7	100.0	100.0
Amantadine	337	329 (97.6)	21.4	96.4	100.0	100.0	100.0
Istradefylline	319	314 (98.4)	41.4	97.8	100.0	100.0	100.0
Zonisamide	650	642 (98.8)	35.5	97.7	100.0	100.0	100.0

PD, Parkinson’s disease; PDC, proportion of days covered; Q, quartile.

Table 5 shows the factors associated with adherence to anti-PD drugs in the all-drugs cohort. The number of anti-PD drugs filled (RR 0.99 per 1 unit increase, 95% CI 0.98–1.00; *p* = 0.010), number of concomitant drugs filled (RR 1.00 per 1 unit increase, 95% CI 1.00–1.00; *p* = 0.032), and prescriptions of anti-PD drugs during hospitalization (RR 0.91, 95% CI 0.89–0.93; *p* < 0.001) were found to be significantly associated with adherence to anti-PD drugs in the all-drugs cohort.

**Table 5:** Factors associated with adherence to anti-PD drugs (PDC) in the all-drugs cohort.

Variable	PDC ≥ 80% *n* = 5,083	PDC < 80% *n* = 163	Risk ratio (95% CI)	*p*-value
Age, years, mean ± SD	77.3 ± 6.8	77.7 ± 7.0	1.00 (1.00–1.00)	0.925
Sex				
Male	2,283 (44.9)	77 (47.2)	1.00 (0.99–1.01)	0.667
Female	2,800 (55.1)	86 (52.8)	reference	
Number of drugs filled, mean ± SD				
Anti-PD drugs	1.9 ± 1.1	2.1 ± 1.2	0.99 (0.98–1.00)	0.010
Concomitant drugs	5.3 ± 3.1	4.8 ± 3.3	1.00 (1.00–1.00)	0.032
Daily levodopa dose at day 0, mg, median (IQR)				
Levodopa	300.0 (300.0–450.0)	400.0 (300.0–525.0)	1.00 (0.99–1.00)	0.198
LED of all anti-PD drugs	420.0 (300.0–632.0)	500.0 (300.0–707.5)	1.00 (1.00–1.01)	0.252
Prescriptions of anti-PD drugs during hospitalization, *n* (%)	865 (17.0)	105 (64.4)	0.91 (0.89–0.93)	<0.001
Comorbidities, *n* (%)				
Depression	694 (13.7)	30 (18.4)	0.99 (0.97–1.00)	0.136
Dementia	1,005 (19.8)	48 (29.4)	0.99 (0.97–1.00)	0.100
Liver failure/renal failure	989 (19.5)	30 (18.4)	1.00 (0.99–1.01)	0.730

CI, confidence interval; IQR, interquartile range; LED, levodopa equivalent dose; PD, Parkinson’s disease; PDC, proportion of days covered; SD, standard deviation.

The results of the sensitivity analysis were broadly similar to those of the primary analysis (Supplementary Tables 7–10). High adherence was observed for each drug, regardless of the calculation method used (PDC ≥80%: sensitivity analysis 1, 92.0–100.0%; sensitivity analysis 2, 91.3–100.0%; sensitivity analysis 3, 75.8–100.0%; sensitivity analysis 4, 75.8–100.0%) (Supplementary Tables 8–10).

## 4 Discussion

This longitudinal descriptive epidemiological study analyzed treatment persistence rates and adherence to drug treatment in Japanese patients with PD and examined the relationship between treatment adherence and comorbidities and polypharmacy during treatment. This study established a cohort for each drug, and to the best of our knowledge, this is the first study to examine persistence rates and adherence to levodopa adjunct medication in Japanese patients with PD.

In this study, persistence rates and adherence to anti-PD drugs used in combination with levodopa were analyzed using a Japanese health insurance claims database, mainly among the elderly. While most previous studies have used hospital databases or corporate health insurance databases, this study includes both hospital and clinic data, which we consider reflects a more representative real-world treatment landscape. We found that the most common new adjuncts to levodopa initiated by elderly Japanese patients with PD during this period were the ropinirole transdermal patch, followed by rasagiline, opicapone, safinamide, and rotigotine. The use of anticholinergics and dopamine agonist immediate-release tablets as adjuncts to levodopa was rare. The prescribing picture was similar to that reported previously in Japan (8).

Overall, more than half of the new anti-PD drugs added to levodopa were discontinued within a year in elderly patients with PD in Japan, and more than half of the patients on zonisamide (59.5%), safinamide (55.8%), droxidopa (55.6%), pramipexole (sustained-release tablet; 53.9%), ropinirole (transdermal patch; 53.9%), opicapone (52.7%), rasagiline (51.9%), and amantadine (51.0%) continued treatment for 1 year without experiencing a 30-day gap period. Adherence was extremely high (>90%) regardless of the anti-PD drug used.

The persistence rates of anti-PD drug treatments in previous studies outside Japan vary (17–21), which may be partly because of differences in the medical history of the target patients and different definitions of treatment persistence. In a previous US database study of patients with PD, 17.2% of all patients continued treatment for 1 year, and persistence was significantly greater in patients who had received prior PD therapy than those who were naive to PD therapy (150.5 vs. 122.7 days, respectively; *p* < 0.001) (17). Higher persistence rates may have been observed in this study because patients with prior PD therapy have a longer duration of continued treatment. By comparison, in a previous Japanese study the 1-year persistence rate for type 2 diabetes medication was 68.0–74.8% (22) and a Japanese database analysis suggested that persistence rates for hyperlipidemia medications were lower in untreated patients (45.0%) than in previously treated patients (77.5%) (23). Although treatment persistence was lower in this study compared with studies of other chronic diseases, the effect of the treatment regimens on treatment persistence was consistent between the studies.

In the multivariate analysis, the factor most strongly associated with non-persistence was the number of concomitant anti-PD drugs, with the exception of inpatient prescriptions. A previous study also showed that the duration of the same treatment regimen is shorter in the later line (21). In the subpopulation of patients taking concomitant anti-PD drugs other than levodopa, differences in persistence rates between drugs were more apparent, with drugs such as zonisamide and safinamide having higher persistence rates. In patients with more advanced PD, the requirement for multiple anti-PD drugs suggests a greater need for short-term drug adjustment, possibly leading to drug discontinuation. The frequency of side effects requiring drug discontinuation in patients with more advanced disease states, along with the effect of wearing-off, may have influenced this trend. Zonisamide and safinamide were less likely to cause side effects leading to discontinuation and may have been more likely to be chosen for continuation, particularly in situations where drug adjustments were necessary. Indeed, these drugs are relatively well tolerated based on network meta-analyses of clinical trials (24, 25). Our treatment persistence results suggest that zonisamide, safinamide, and opicapone were better balanced than other drugs in the same class in terms of efficacy and safety. Another reason might be the number of daily doses, as within each drug class (dopamine agonists, catechol-O-methyltransferase inhibitors, and monoamine oxidase B inhibitors), drugs with a once-daily dosing frequency had higher persistence rates than drugs that could be dosed more than once a day. The results show that the persistence rates of zonisamide and safinamide remain high due to their clinical benefits, despite these drugs being among the most expensive in Japan. This suggests that the influence of the cost of medications may be relatively small compared with other factors in this study.

In the present study, the comorbidity of depression was also associated with non-persistence (Table 3). One possible explanation is that depression was a barrier to regular visiting behavior, as such symptoms are recognized to impact on the self-management of chronic illnesses (26, 27), including PD (28). Another factor that may inhibit the realization of stable treatment may be the narrowing of treatment options for PD in patients with depression, as the concomitant use of monoamine oxidase B inhibitors, one of the major anti-PD drug classes, with antidepressants is contraindicated in such patients. Other factors found to be associated with non-persistence in the present study were older age and female sex (Table 3). It is assumed that older PD patients had a faster disease progression (29) and women have a higher risk of developing motor fluctuations and dyskinesia (30). Additionally, in general, elderly patients are more prone to drug-related side effects because of impaired liver and kidney function. Therefore, these patients may be more likely to require a drug change.

While treatment adherence to treatment with anti-PD drugs was extremely high in the present study, we conducted an exploratory analysis of factors associated with poor adherence, which has been identified as a challenge for PD treatment in previous international studies (31–33). A review of clinical studies involving non-Japanese patients with PD found a maximum non-adherence rate of 67% (5). However, in a recent retrospective cohort study in the US, the median PDC ranged from 85.7 to 91.2% (21). In contrast, the present study showed more favorable results, as the proportion of patients with good treatment adherence (PDC ≥80%) was approximately 96.9%, and the median PDC for all drugs was 99.7%. One possible reason for the discrepancies between the present study results and previous studies may be the difference in adherence measures. However, as in several other studies, PDC and MPR were also calculated with the denominator being the last fill date in the follow-up period, and adherence was still very high (Supplementary Tables 9, 10). Other reasons may be differences in study design, patient background characteristics, and cultural differences. The present study generally reflects the current situation of Japanese patients with PD better than other patient surveys in terms of average patient age (3) and medical facilities, because the claims database that is mainly used in Japanese studies does not include data on prescriptions for the elderly and clinics. Another possible explanation is that adherence is higher for drugs used after other treatments than for drugs used for the first time to treat the disease, as has been frequently observed in a previous study (17). This study included drugs used after the initiation of oral levodopa therapy, and it is assumed that adherence was higher than in analyses covering drugs from all lines of therapy. For patients who have already been taking levodopa multiple times a day, taking levodopa adjunct drugs (often taken once a day) is not considered burdensome. Such patients are also less likely to drop out as a result of dopaminergic side effects because most levodopa adjunct drugs exert dopaminergic effects. In Japan, the proportion of patients with good adherence (PDC ≥80%) to other chronic disease medications was 80–90% (34–38). In comparison, adherence to anti-PD drugs in this study was high, possibly because, unlike other chronic diseases, PD substantially affects daily life because of the occurrence of motor symptoms and wearing-off. Accordingly, poor adherence has a notable negative impact on daily life for people with PD, and this may be one reason for the high level of adherence. Furthermore, this study analyzed prescription data and evaluated adherence to treatment rather than adherence to medication. Some patients who regularly visit the hospital may receive a certain amount of prescription medications despite having residual medications at home, which may lead to overestimation of medication adherence.

In a previous longitudinal Japanese study evaluating treatment adherence to istradefylline in patients with PD, factors associated with the likelihood of lower adherence were the use of fewer anti-PD drugs, higher prevalence of anxiety/mood disorders, and higher prevalence of mild cognitive impairment/dementia (9). Similar to the previous Japanese study, in the present study, the number of prescriptions for anti-PD drugs was identified as a factor associated with poor adherence, but the effect was minor. Factors identified in previous studies such as older age, cognitive decline, depression, and treatment complexity due to comorbidity treatment (5, 7, 9) did not show statistically significant associations in the present study. As for the lack of impact of cognitive decline, given that many of the participants in this study were elderly, it is possible that family members or caregivers managed their medications. However, information on caregiving practices is unavailable in the Japanese health insurance claims database that we used. Statistical power may have been insufficient owing to the small number of non-adherent patients.

The present study has some limitations. It was based on a claims database and could not assess whether the patient actually took the prescribed treatment. For drugs that require titration, the lack of information regarding specific dosing instructions is a limitation. Additionally, no information was provided about patients’ clinical symptoms, the severity of PD, the presence of motor fluctuations (e.g., wearing-off, dyskinesia), cognitive function status, changes in these items after starting to use index drugs, the duration of the disease, drug side effects, medication management by caregivers, changes in concomitant medications after starting the index drug, or reasons for discontinuation of treatment, which may lead to confounding. Claims data could also be subject to bias related to data entry and processing. Specifically, the lack of validation studies for PD diagnosis represents a potential limitation. Furthermore, the number of prescription days at discharge was supplemented. Although the presence or absence of inpatient prescriptions was identified as a factor associated with persistence rates and adherence, the above supplementation process may have influenced the persistence and adherence in patients who experienced hospitalization. Owing to the lack of adjustments for patient background characteristics leading to confounding and limitations of the database, statistical comparisons between drug cohorts were not possible. The study followed patients for 1 year. Side effects such as dopamine dysregulation syndrome appear with a long-term administration, and a study with a longer observation period is required to address this. Lastly, it should be noted that this study included anti-PD drugs such as zonisamide and droxidopa, which have received regulatory approval for the treatment of PD only in Japan.

In conclusion, in Japanese patients with PD, more than half of the new anti-PD drugs added to levodopa were discontinued within 1-year, and adherence to treatment assessed by filling records was shown to be extremely high. Treatment persistence varied by drug, with zonisamide and safinamide having relatively high rates. The number of concomitant anti-PD drugs was strongly associated with treatment persistence for anti-PD drugs. However, this study was based on claims data, so it may not accurately reflect patients’ actual dosing status, and further research is needed to validate these findings.

## Data Availability

The data analyzed in this study was obtained from IQVIA Solutions Japan G.K., but were used under license for the current study; therefore, restrictions apply and the data are not publicly available. The original contributions presented in the study are included in the article/Supplementary material, further inquiries can be directed to the corresponding author.
