# Supplementary material for: Persistence and adherence to levodopa adjunct medications in elderly patients with Parkinson’s disease: a retrospective cohort study using a Japanese claims database
Source: Front Neurol. 2025 Apr 10;16:1560431. doi: 10.3389/fneur.2025.1560431 (PMC12018396; doi:10.3389/fneur.2025.1560431)
Supplement: Supplementary file 2 [file Table_2.docx]

Supplementary Material

## Supplementary Figure


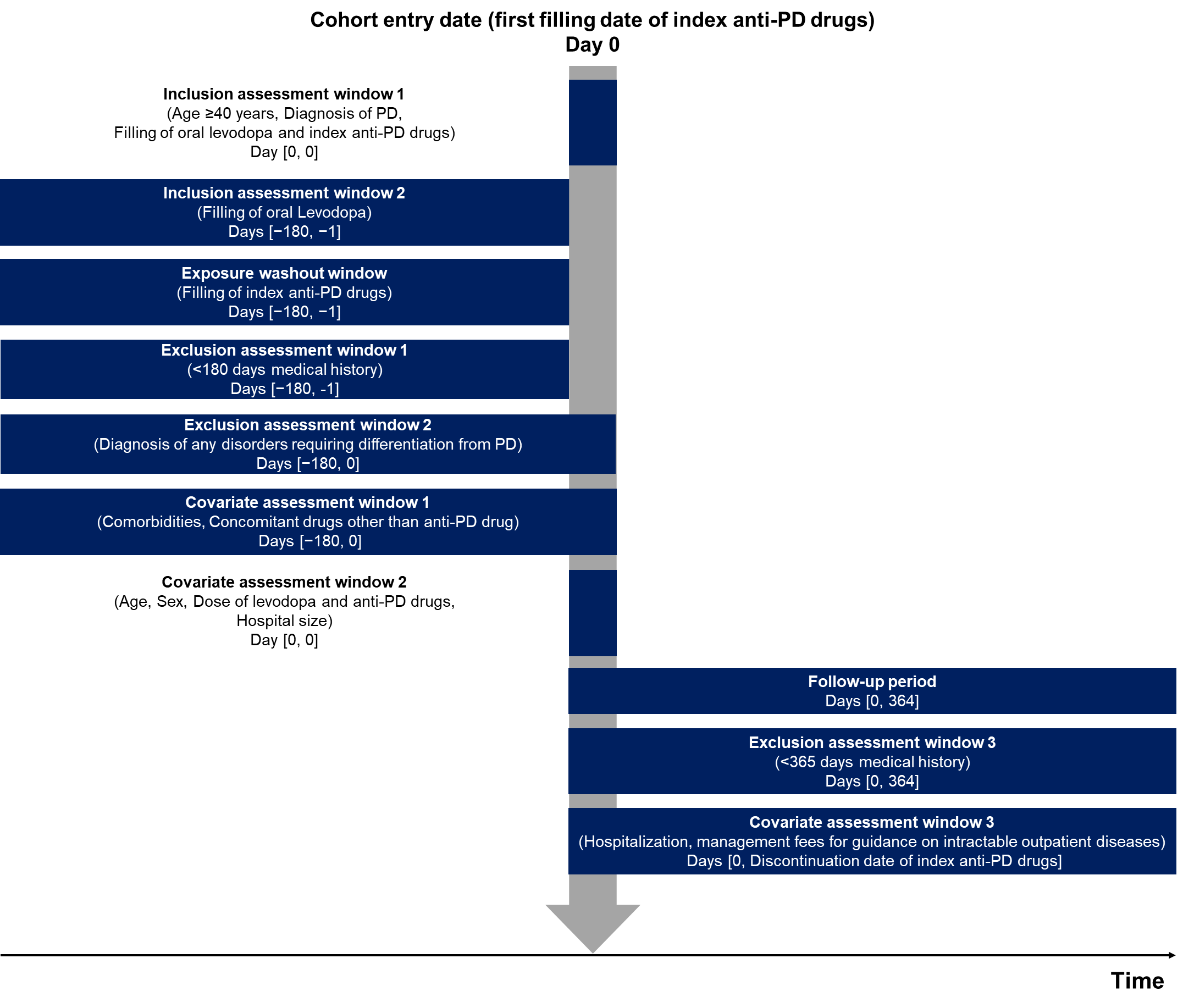


**Supplementary Figure 1.** Study design

Abbreviation: PD, Parkinson’s disease.

**Supplementary Tables**

**Supplementary Table 1.** Drug codes

**Supplementary Table 2.** Disease codes

**Supplementary Table 3.** Background characteristics of the study population by individual drug

**Supplementary Table 4.** Study population in each cohort

**Supplementary Table 5.** Subgroup analysis of persistence of anti-PD drug treatment

**Supplementary Table 6.** Subgroup analysis of adherence to anti- PD drugs

**Supplementary Table 7.** Sensitivity analysis of persistence of anti-PD drug treatment

**Supplementary Table 8.** Sensitivity analysis of PDC of anti-PD drug treatment

**Supplementary Table 9.** PDC of anti-PD drug treatment to the last fill date in the follow-up period

**Supplementary Table 10.** MPR of anti-PD drug treatment to the last fill date in the follow-up period
